# The Relationships between the Population Density of Fir Bark Beetles and Niche Breadth

**DOI:** 10.3390/insects15060422

**Published:** 2024-06-05

**Authors:** Andrzej Borkowski

**Affiliations:** Department of Environmental Biology, Institute of Biology, Jan Kochanowski University, Uniwersytecka 7 Str, 25-406 Kielce, Poland; andrzej.borkowski@ujk.edu.pl; Tel.: +48-22-349-63-19

**Keywords:** *Cryphalus piceae*, *Pityokteines curvidens*, *P. spinidens*, *Abies alba*, resource partitioning, niche breadth, nonlinear models

## Abstract

**Simple Summary:**

Determining the ecological niche breadth of various organisms is a key objective in contemporary biological research. Studies of this type play a significant role in describing biotic interactions, which is becoming particularly important in the context of observed climate change. A method has been developed that enables the monitoring of the impact of climatic changes at the local level, in specific tree stands. The statistical foundations of the method enable the computation of errors of estimation. The method offers a high precision and explains approximately 80% of the variation in the niche breadth of bark beetles on natural traps, while the mean relative errors of estimation do not exceed 20%. The niche breadth parameter obtained from the derived regression equations may be used in models that describe—for example—the impact of observed climate change on the population dynamics of bark beetles. The results suggest a need for further research to derive regression equations for firs growing in different regions of Europe.

**Abstract:**

Bark beetles are a significant link in the chain of diseases that lead to the accelerated dying of firs (*Abies alba* Mill.), a key species in the cultivation of stable mixed-tree stands. The aim of this work was to evaluate biotic interactions in populations of bark beetles that colonised natural traps made from firs. The tested hypothesis was that the niche breadth of the species increases with the increasing density of the population. The research was carried out in near-natural forests containing fir, growing in the Suchedniów-Oblęgorek Landscape Park in central Poland. Data were collected from 30 traps trees and 30 windfalls in the years 2010–2023. *Cryphalus piceae* Ratz. prefers heavily weakened trees, as shown by the fact that it colonised all of the natural traps, which lack any defensive reactions. The sampling method used in the study proved effective, as confirmed by the segregation of the niches of all of the bark beetles. Using nonlinear regression (linearisable model and piecewise linear regression), models were constructed that describe the niche breadths of the bark beetles. The niche parameter is correlated with the density of colonisation. The derived models explain around 77–84% of the variation in the niche breadth of bark beetles on natural traps. The mean relative errors of estimation do not exceed 20%. The niche breadth parameter obtained from the derived regression equations may be used in models that describe—for example—the impact of observed climate change on the population dynamics of bark beetles.

## 1. Introduction

The niche is an important concept in ecology, evolution, and conservation biology. A review of the literature [1,2] found that it is still largely unknown how the ecological niche breadth evolves, particularly how and how quickly the niche breadth expands. Field studies of how the niche breadth evolves are essential for providing mechanistic details and allowing the development of a comprehensive theory and improved prediction of biological responses under global change [1]. The niche breadth parameter can be evaluated on a global scale [3] or locally, in a specific tree stand [4]. A possible reason for the relatively small number of studies on bark beetles [5,6,7,8,9,10,11] is the large amount of work that they demand, and the associated economic costs. Such research requires the precise removal of bark from whole trees, combined with the simultaneous marking of egg galleries. This work is highly invasive, which severely restricts the possibilities of evaluating the niche breadth in protected areas.

Silver fir (*Abies alba* Mill.) is a key species in the cultivation of stable mixed-tree stands [12], and is an economically important coniferous tree [13]. For approximately 200 years, the gradual disappearance of fir from stands has been observed in Europe. The problem of fir regression is a complex one [14,15,16], but one of the main causes is reported to be the lack of adaptability of populations due to the low genetic variation [17,18,19]. There is also a wide discussion on the effect of observed climate change on the adaptive potential of fir [20]. Irrespective of differences in the assessment of the ecological plasticity of this species [12,21,22,23,24], the health of firs is impaired mainly under conditions of persistent water stress [25,26].

Among other organisms, weakened fir trees are attacked by bark beetles, chiefly *Cryphalus piceae* Ratz. and the species of the genus *Pityokteines* Fuchs [15]. These insects are a significant link in the chain of diseases that accelerate the dying of firs throughout their range [27,28,29,30,31]. In the case of favourable conditions of reproduction and a high population density, they may also attack healthy trees [32]. Hypotheses regarding the dieback of fir in the last century in Poland were described by Ukleja-Dobrowolska [33]. It is particularly visible in the Świętokrzyskie (Holy Cross) Mountains [34], where firs occur close to the northern limit of their natural range [35]. In more open stands, there are large numbers of windfalls, which provide breeding material for bark beetles [36].

Changes in climate parameters have both direct and indirect effects on insects [37,38,39,40]. Generally, in case of a significantly faster rate of reproduction, bark beetles adapt to new conditions more rapidly than the host trees. In addition to abiotic conditions, the population dynamics of forest insects are strongly affected by biotic interactions, including intraspecific and interspecific competition [41]. However, the combined impact of these factors, as well as their interactions, are still not well-understood [42,43,44]. In models that take into account biotic variables (competition, natural enemies, or phenotype), the proportion of unexplained variation may drop from 60% to below 30% [45,46,47,48]. Studies of eruption insects suggest that the main regulatory factors acting on non-outbreak and outbreak populations may differ significantly [49,50,51]. There is a clear lack of knowledge about the impact of a whole range of biotic variables on bark beetle population dynamics [39]. Additionally, for all eruptive insect model systems, it remains important to investigate how climate change alters insect biotic interactions and potentially the dynamics of the entire system [39,52].

It can be expected that intra- and interspecific competition would, respectively, expand and constrain the population niche breadth [53]. However, a review of the literature [54,55] indicates that this relationship is not as clear as had been assumed, and that further work is needed to extend what has been a simplistic but useful conceptual construct.

The goal of the present study was to describe the biotic interactions relating to bark beetles occurring on fir. The tested hypothesis was that the niche breadth increases with increasing population density.

The niche breadth parameter calculated from the derived regression equations may be used in models that describe, for example, the impact of climate factors on the population dynamics of bark beetles.

In addition, the effect of the type of the breeding material on the colonisation of *A*. *alba* by bark beetles was evaluated.

## 2. Materials and Methods

### 2.1. Study Area

The research was carried out within the Suchedniów-Oblęgorek Landscape Park (S-OLP) at 50°55′ N 20°45′ E, 200–400 m above sea level. The main resource of this Park is forests growing on fertile land, which occupy 91% of its area. The great majority of these are remnants of the former Holy Cross Primeval Forest (Puszcza Świętokrzyska). The Park’s forests contain all main tree species, with the largest contributions to the tree population coming from pine (*Pinus sylvestris* L., 50.2%), fir (*Abies alba* Mill., 26.6%), and beech (*Fagus sylvatica* L., 10%). Fir and beech occur here at the northern boundary of their range. The climatic conditions within the Park are characteristic of a mountain climate [56].

**Figure 1:**
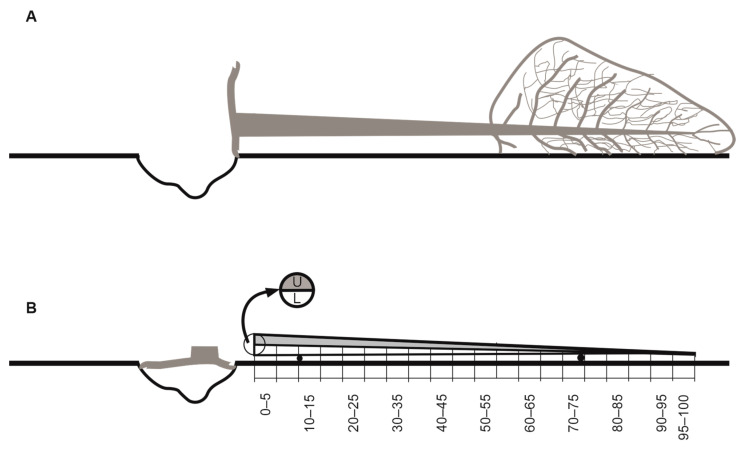
(**A**) *Abies alba* windfall (**B**) used as a trap tree; letters *U* and *L* indicate the upper and lower section of the trap tree, respectively. Numbers 0–5, ..., 95–100 indicate the percentage units of the stem, respectively.

For the purposes of the study, stands containing fir were selected, distributed uniformly throughout the area of the Park. The maximum distance between stands containing fir trees was 1 km [57]. Data were collected in the years 2010–2023. Field inspections of the stands were carried out at the end of March each year, the aim of which was to select four fir windfalls with undamaged stems that had fallen in March (a total of 60 windfalls). Two of these were cut off from their roots, and branches were removed from their stems (a total 30 trees). The stems were then placed on supports made from a different species of tree, with a thickness of 20 cm (Figure 1A).

The trees prepared in this way will henceforth be referred to as “trap trees”. Such trees are used in the monitoring of bark beetles [58]. The remaining two windfalls were left without intervention until the time of the counting of bark beetle egg galleries in July. Before the entomological analysis, the roots and branches of the windfalls were cut from the stems (Figure 1B). The following operations were performed on each stem:

- Measurement of stem length and the diameters at the thicker and thinner ends. The average diameters at the thicker end for the trap trees (35.8 ± 6.9 SD) and the windfalls (33.7 ± 7.3 SD) were similar (*t*-test: *t* = 1.1109; *df* = 58; *P* = 0.2712). The average length of the trap trees (22.1 ± 1.96 SD) was greater than that of the windfalls (20.6 ± 2.5 SD) (*t*-test: *t* = 2.6780; *df* = 58; *P* = 0.0096);
- Division of the stems into units each covering 5% of the stem length (20 equal units). For each unit, the diameters at the thicker and thinner ends were measured;
- Division of the stems lengthwise into upper and lower sections.

In total, each stem was divided into 40 sections (20 units, two sections on each). Bark beetle egg galleries were counted separately on the bark of each section. To avoid damage to the bark during its removal from the stem, on each unit cuts were made on the circumference, the sides, and the upper and lower parts of the stem. Therefore, for each stem unit, four pieces of bark were collected. The infestation of individual stem sections was determined by counting (1) the number of nuptial chamber *C. piceae*, and (2) the number of egg galleries *P. curvidens* and *P. spinidens*.

- The nuptial chamber of *C. piceae* is approximately 0.5 cm long. Females deposit eggs in clusters. A female lays from 20 to 40 eggs. After hatching, larvae excavate 2–4 cm-long maternal galleries in all directions from the nuptial chamber [59].
- Egg gallery of *P. curvidens*: Males excavate nuptial chambers, from which females excavate two maternal galleries upward and downward. Later, these maternal galleries are divided into two arms to the right and to the left. Maternal galleries are about 5–10 cm long and 1 mm wide. They are mostly in the bark, but also visible on the wood [59].
- Egg gallery of *P. spinidens* is “star”-shaped. Maternal galleries (usually 3–8) are about 1 mm wide and 10 cm long. They start from the nuptial chamber and bend in a distance not shorter than 10 mm (egg gallery). Pupal chambers are in the sapwood or between the sapwood and bark [59].

The total density of bark beetle colonisation on each stem was calculated based on the number of egg galleries in all sections and the lateral surface area. The lateral surface area was calculated using the formulae given by Borkowski [11]. To equalise the real distributions of egg galleries on the stems, the method of distance-weighted least squares was applied [60].

### 2.2. Measures Used to Describe the Niche Breadth Parameters of Bark Beetles

#### 2.2.1. Levin’s Measure of Niche Breadth [61]

$$B_{i}=1/\sum_{h=1}^{n}p_{ih}^{2}$$

where *p_ih_* is the proportion of species *i* in section *h*, *h* denotes a stem section covering half the circumference of a tree unit, and *n* is the number of sections.

The niche breadth *B* was standardised to a scale from 0 to 1 [62]:

$$B_{i}^{\prime }=\frac{B_{i}-1}{n-1}$$

The niche breadth index takes the value 1 if the use of the resources by a species is uniform along the entire length of the stems. The differences in the niche breadths of bark beetles on natural traps were compared using the Kruskal–Wallis test [63].

#### 2.2.2. Index of Niche Overlap (Morisita’s Index) [64]

$$\hat{C}=\frac{2\sum_{h=1}^{n}p_{ih}\cdot p_{jh}}{\sum_{h=1}^{n}p_{ih}\left[\frac{n_{ih}-1}{N_{i}-1}\right]+\sum_{h=1}^{n}p_{jh}\left[\frac{n_{jh}-1}{N_{j}-1}\right]}$$

where *p_ih_* and *p_jh_* are the proportions of species *i* and *j* in section *h*, *n_ih_* and *n_jh_* are the numbers of individuals of species *i* and *j* in section *h*, and *N_i_* and *N_j_* are the total numbers of individuals of species *i* and *j* on the stem.

Differences between the niche overlaps of bark beetles were tested using the *t*-test for independent samples [63].

#### 2.2.3. Proportional Similarity Index (PSi) [65]


$${PS}_{i}=1-0.5\sum_{h=1}^{n}\left|p_{ih}-p_{jh}\right|$$

A value of *PS_i_* < 0.7 indicates segregation of the niches of bark beetle species [66]. To evaluate the difference between theoretical and empirical values of *PS_i_*, the one-sample *t*-test was used [63].

### 2.3. Models for Bark Beetle Niches

The explanatory variables for the construction of the model represented parameters of colonisation (number of egg galleries/m^2^) and features of the stems (stem diameter in bark at the thicker end; diameter at breast height). Analysis of scatterplots for pairs of variables showed the dependence of niche breadth is dependent on the density of colonisation of stems by the beetles. These relationships are of curvilinear type. The nonlinear model for *C. piceae* was brought to a linear form by means of a logarithmic transformation (Equation (1)):

(1) $$B_{Cp}^{\prime }=a_{0}+a_{1}\times lnD_{Cp}$$

Piecewise linear regression (PLR) was used to describe the niche breadths of *Pityokteines curvidens* Germ. (Equation (2)) and *Pityokteines spinidens* Reitt. (Equation (3)):

(2) $$B_{Pc}^{\prime }=\left(a_{0}+a_{1}D_{Pc}\right)\left(D_{Pc}\leq \alpha_{1}\right)+\left(b_{0}+b_{1}D_{Pc}\right)(D_{Pc}>\alpha )$$

(3) $$B_{Ps}^{\prime }=\left(a_{0}+a_{1}D_{Ps}\right)\left(D_{Ps}\leq \alpha_{1}\right)+\left(b_{0}+b_{1}D_{Ps}\right)(D_{Ps}>\alpha )$$

where $B_{Cp}^{\prime },\;B_{Pc}^{\prime },\;B_{Ps}^{\prime }$ are the niche breadths of *C. piceae*, *P. curvidens*, and *P. spinidens*; *a*_0_ and *a*_1_ are parameters of the model; *ln* is natural logarithm; *D_Cp_*, *D_Pc_*, and *D_Ps_* are the total densities of colonisation of stems by *C. piceae*, *P. curvidens*, and *P. spinidens*; and *α* is the breakpoint.

To find the minimum of the loss function, the method of quasi-Newton estimation was used [67]. The fit of the models was determined on the basis of the adjusted coefficient of determination ($R_{adj}^{2}$) and the root mean square error (RMSE) [68,69]. The distribution of homoscedasticity of regression residuals and their conformance to normal distribution were analysed using White’s test [70] and the Shapiro–Wilk test [63].

## 3. Results

### 3.1. Colonisation of Firs by Bark Beetles

A total of 374,770 egg galleries of all of the major species of bark beetle was counted on the fir stems. *C. piceae* was the dominant species (*n* = 353,521 egg galleries, 94.20% of the total) and had colonised all of the fir stems. This species co-occurred mainly with bark beetles of the genus *Pityokteines* Fuchs. The frequencies of occurrence of *P. curvidens* on the trap trees and windfalls were, respectively, 57% and 90% (*n* = 12,121 egg galleries, 3.23% of the total), and those of *P. spinidens* were 37% and 80% (*n* = 9128 egg galleries, 2.43% of the total). Other species—*Trypodendron lineatum* L. and *Pissodes piceae* III.—were found with a very low frequency (20.0 and 16.7% of firs, respectively), together accounting for fewer than 0.2% of all egg galleries identified in the stems.

**Figure 2:**
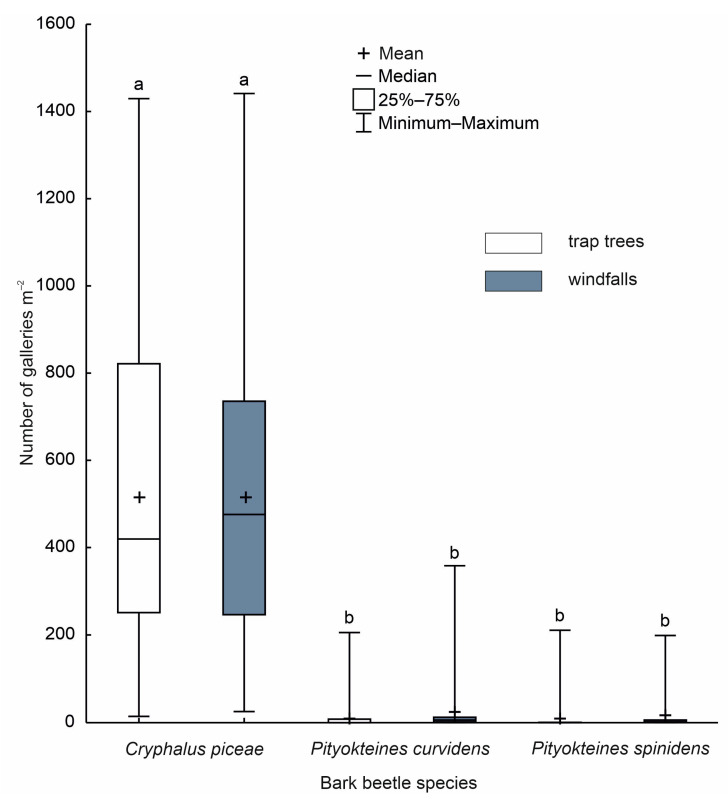
Bark beetle attack density; a, b: median marked with different letters indicate statistically significant differences among the bark beetle species (Kruskal–Wallis ANOVA; *P* < 0.001).

The differences in the levels of colonisation of trap trees and windfalls by the beetles were not significant (Kruskal–Wallis ANOVA: *H_df_*_= 5_ (*n* = 180) = 121.8434; *P* < 0.001; Figure 2).

**Figure 3:**
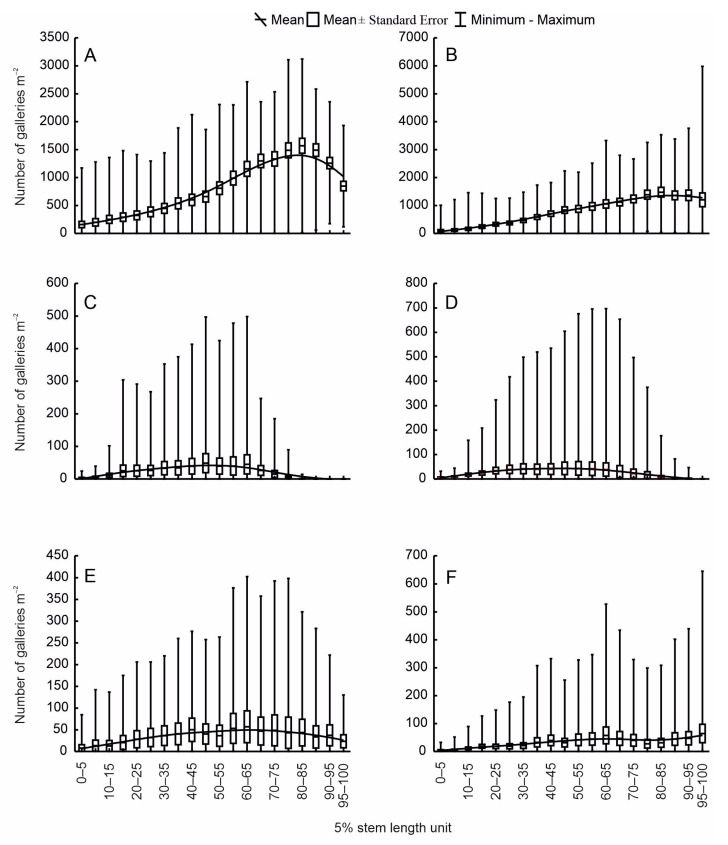
Colonisation patterns by: *Cryphalus piceae* (**A**) trap trees and (**B**) windfalls, *Pityokteines curvidens* (**C**) trap trees and (**D**) windfalls, and *Pityokteines spinidens* (**E**) trap trees and (**F**) windfalls; solid line—smoothed infestation density (distance-weighted least squares).

*C. piceae* and *P. spinidens* colonised stems along their entire length, but mainly on the thinner part (Figure 3A,B,E,F). *P. curvidens* colonised mainly the middle part of stems, excluding the tops (Figure 3C,D).

The curve of the distribution of bark beetle egg galleries on the stems indicates a fall in density with increasing distance from the section with maximum colonisation. The distribution curve of *P. spinidens* egg galleries on windfalls exhibits two local extrema (Figure 3F). *C. piceae* prefers the lower part of stems (proportion *p* = 0.62; one-sample *t*-test: *P* < 0.05). *Pityokteines* species prefer the upper part of stems of trap trees (*p* = 0.70–0.72; one-sample *t*-test: *P* < 0.05), but do not display a similar preference on windfalls (one-sample *t*-test: *P* > 0.05).

### 3.2. Biotic Interactions

The niche breadth of *C. piceae* (Levins’ measure: 0.56–0.57) was 2–3 times greater than that of *Pityokteines* species (Levins’ measure: 0.17–0.25) (ANOVA: *F*_5,133_ = 20.8816; *P* < 0.001; post hoc Tukey LSD test). The differences in bark beetle niche breadths on trap trees and windfalls were not significant.

Irrespective of the type of breeding material, the two species of *Pityokteines* co-occurred on stems more frequently (Kruskal–Wallis ANOVA: *H_df_*_=5_ (*n* = 113) = 40.1342; *P* < 0.001). The degree of coexistence (Morisita’s index: 0.67) was approximately 3–5 times greater than in the case of resources shared with *C. piceae* (Morisita’s index: 0.13–0.25). The differences in the niche overlap of bark beetles on trap trees and windfalls were not significant (*t*-test: *P* > 0.05). The niches of the beetles were segregated. The values of the proportional similarity index (*PS_i_*) calculated for pairs of species (0.12–0.46) were below 0.7 (one-sample *t*-test: *P* < 0.05).

**Figure 4:**
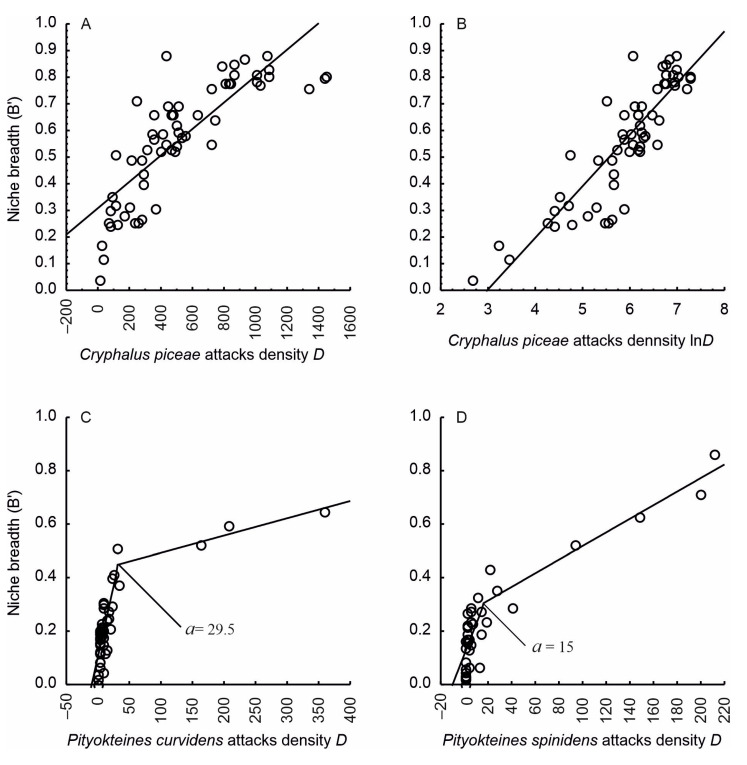
Relationship between bark beetle attack density (number of egg galleries/m^2^) and niche breadth; (**A**,**B**)—*Cryphalus piceae*, (**C**)—*Pityokteines curvidens* and (**D**)—*Pityokteines spinidens*; *a*—breakpoint.

The results of the regression analysis indicate that there exist statistically significant dependences between the niche breadth (*B’*) and the density of colonisation of stems by bark beetles (*D*). This relationship is nonlinear (Figure 4). By means of a logarithmic transformation of the density of *C. piceae* colonisation (*lnD*), it was possible to bring the nonlinear regression model (Figure 4A; Equation (4)) to a linear form (Figure 4B; Equation (5)):

(4) $$B_{Cp}^{\prime }=0.3082+0.0005\times D$$

(5) $$B_{Cp}^{\prime }=-0.5786+0.1939\times lnD$$

The values of model coefficients and their statistical evaluation for particular variables are shown in Table 1. Following transformation, all points lay along a straight line (Figure 4B), which improved the fit of the model ($R_{adj}^{2}\;$= 0.7669). All parameters of the model are significant (*P* < 0.001). The mean relative error of estimation is 18.7%. The positive value of the directional coefficient indicates that the niche breadth increases with the increasing density of the colonisation of stems by *C. piceae*.

For both species of *Pityokteines*, the dependence between the niche breadth and population density exhibits an abrupt change (Figure 4C,D). The niche breadth initially rises rapidly, but, above a certain point, the growth becomes slower. Based on a scatterplot analysis using logical operators, two separate linear regression equations were derived for each of *P. curvidens* (Equation (6)) and *P. spinidens* (Equation (7)):

(6) $$B_{Pc}^{\prime }=\left\{\begin{array}{c} \;0.1028+0.0106D\;for\;D\leq 29.5\;\\ \;0.4274+0.0006D\;for\;D>29.5\; \end{array}\right.$$

(7) $$B_{Ps}^{\prime }=\left\{\begin{array}{c} \;0.1126+0.0113D\;for\;D\leq 15\;\\ \;0.2629+0.0025D\;for\;D>15\; \end{array}\right.$$

The calculated value of the breakpoint of the regression line is 29.5 for *P. curvidens* and 15.0 for *P. spinidens*. The values of the adjusted coefficient of determination ($R_{adj}^{2}$) exceed 80% (Table 1), which means that the constructed regression models with the determined breakpoints fit well to the observed data. The directional coefficients differ from zero and take positive values. The total loss function takes similar values: 0.1838 for *P. curvidens* and 0.2055 for *P. spinidens*.

Following the calculation of the parameters of the models, the residual values were analysed. The residual variance in the constructed models is constant, and the residual distribution conforms to a normal distribution (Table 2). The residual properties indicate that the models are correctly constructed.

## 4. Discussion

### 4.1. Tree Infestation

The physiological condition of trees is a key factor determining the species composition of the bark beetle populations that colonise them [71,72]. The species *C. piceae* prefers heavily weakened trees, in contrast to the species of the genus *Pityokteines* [73]. For this reason, all of the natural traps, which lack defensive reactions, were colonised by *C. piceae*, and some of them exclusively by that bark beetle species. The fact that *Pityokteines* occurred almost twice as frequently on the windfalls may indicate differences in the suitability of natural traps as food sources. Due to the partially preserved contact between the tree roots and the ground, windfalls maintain moisture in the phloem tissue for a longer time. For example, pine and spruce windfalls may be colonised in the next growing season [74,75,76]. We should exclude the possibility that the reason for the low density of the colonisation of natural traps by *Pityokteines* species in the S-OLP area was a low species population. Studies carried out in adjacent stands that had been weakened to differing degrees [77] showed that insect migrations at swarming time could lead to the colonisation of heavily weakened firs, provided that the conditions were suitable for them. Among bark beetles, the dispersal and flight behaviour are relatively well-studied in the case of *Ips typographus* L. [78] recaptured marked specimens at distances of up to 1600 m, and Duelli et al. [79] found specimens at distances of up to 500 m. A study by Botterweg [80] showed that the beetles are capable of flying a distance of up to 750 m in forests over the course of one day and found single specimens at a distance of 8 km from the forest. Adult *T*. *piniperda* L. may disperse over a distance of several kilometres during their spring flights, even over water, in search of host materials [81,82,83].

A comparison of the density of colonisation of stems by *C. piceae* in the S-OLP area (more than 500 egg galleries/m^2^; Figure 2) and in heavily weakened stands in the Ojców National Park (550 egg galleries/m^2^) [77] confirms the continuing weakening of firs in the Holy Cross Mountains (Góry Świętokrzyskie) [84]. In stands where fir grows in optimum habitat conditions, the colonisation of stems is much lower, at approximately 98 egg galleries/m^2^ [85]. The above data indicate the significant role of *C. piceae* in accelerating the process of dying of fir stands in the Holy Cross Mountains. The similar level of colonisation of natural traps by *C. piceae* means that windfalls can be used to limit population numbers of this species. In the Holy Cross Mountains, this is a breeding material that occurs frequently and in large quantities [36].

### 4.2. Biotic Interactions

The sampling method adopted in this work is effective, as evidenced by the segregation of the niches of all bark beetles. This method is based on the assumption that an unspecified factor conditioning the colonisation of stems by bark beetles acts proportionally along the whole of the stem length and circumference. The factors used for the segregation of bark beetle niches have not always been fully effective. For example, bark thickness was not an effective factor for the segregation of bark beetle niches on spruce [6] or pine [7]. The sharing of a single ecological niche seems only to be an apparent phenomenon, and probably results from the insufficient precision of the adopted segregation factor. The infestation of stems may be irregular, as is confirmed by the local extrema in the colonisation of windfall stems by *P. spinidens* (Figure 3F) [36]. In such cases, or when there occur multiple species with similar ecological requirements, it is necessary to increase the precision of the segregation factor. For example, the length of the units into which the stem is divided may be reduced from 5% to 2.5% of the stem length (to obtain 40 equal parts instead of 20), and the number of sections in each unit may be increased from two to four (an upper and lower section and two side sections).

The study has confirmed the hypothesis that an increase in the population density leads to a larger niche breadth. Other potential factors have been excluded, such as the effect of the beetle’s body size [86] or the swarming date [7,87]. *C. piceae*, being the smallest beetle, could be expected to occupy the narrowest niche [86]; moreover, in the S-OLP area the bark beetle species swarm at similar times [88]. For the *Pityokteines* species, the relationship of the niche breadth with population density exhibits an abrupt change. At a low population density, the lack of competition means that the insects settle on stems over the entire range of their feeding optimum. However, above a certain threshold density (Figure 4C,D), competition increases, which causes some insects to colonise less preferred stem resources (Figure 3). Bark thickness may be a factor that limits the colonisation of the thickest part of a fir stem. It is a mechanical barrier that makes it more difficult for the beetles to burrow through to the phloem. A similar phenomenon has been observed in the case of *Tomicus piniperda* L., among others; for this species, pine bark with a thickness of over 30 mm presented a barrier to colonisation [89,90]. The dependence of the niche breadth on population density has been observed both for bark beetles [7,11] and for other groups of organisms [91,92,93].

The derived models explain around 77–84% of the variation in the niche breadth of bark beetles on natural traps. As the research indicates, increasing the precision of the method is possible, among other things, by including the bark thickness in the equation [6,7]. This parameter is a significant factor in the niche segregation of most species found on pine and spruce. Another factor in niche segregation could be stem moisture. The significant role of this parameter may be indicated by studies in which the precision of the segregation factor was increased from one (division of the stem into equal parts along the longitudinal axis) to two (division of the stem into equal parts along the longitudinal and transverse axes) dimensions [10]. In the first case, no niche segregation was found for *Pityogenes bidentatus* Herbst. and *Hylurgops palliatus* Gall. It could be related to the ecological specialisation regarding the moisture content of the breeding material of the two bark beetle species. In all the trap trees, egg galleries of *H. palliatus* were found to occur only in the lower sections, which is consistent with the ecological requirements of this species. *H. palliatus* is a hygrophilous organism, principally occurring in shaded and moist environments, and colonising stems along their entire length excluding branches. In contrast, *P. bidentatus* colonises stems along the entire circumference including branches [88].

The niches of bark beetles colonising fir stems are segregated, which suggests differences in the food preferences of these insects. Interspecific competition is minimised by means of spatial specialisation [94]. *C. piceae* and *P. spinidens* occur in the thinner part of stems, and *P. curvidens* in the central part [88]. Moreover, differences in the colonisation of the upper and lower parts of stems by particular species indicate that there are differences in their requirements regarding moisture. *C. piceae* prefers the lower part of the stem, where the moisture may be higher, while the *Pityokteines* species select the dryer upper part. The uniform colonisation of the circumference of the surface of windfall stems by species of *Pityokteines* results from the fact that they are situated higher above the ground. Unlike in the trap trees, the lower part of the stem loses moisture more rapidly, which favours its colonisation by these species.

In constructing models, data collected in natural and near-natural forests are of particular value. For this reason, methods being developed for measuring the niches of bark beetles should ensure the lowest possible degree of interference with the forest ecosystem. The fact that all of the windfalls were infested indicate that these may be successfully used as traditional traps instead of felling live trees, both in scientific research and in forestry.

The derived regression equations may be useful in constructing models that simulate the reactions of bark beetles to observed climate change. In spite of the wide study of bark beetles, data for only six species have been used to develop climate-driven phenology and ecosystem models [38]. The only species occurring in Europe for which such models have been constructed is *I*. *typographus*. Population densities can be estimated using the previously developed method for computing the density of bark beetle colonisation on fir windfalls [36,84,95]. The statistical foundations of this method enable the calculation of errors of estimation. It is also a low-invasive method, as it requires the removal of bark from only two-metre-long sections of stems, and it can, therefore, also be used in areas subject to strict protection.

Because the experiments were carried out over many years, the method may be expected to be more resistant to the effects of abiotic and biotic factors, which is of particular importance with regard to concerns about the possible influence of observed and forecasted changes in climatic conditions—particularly global warming—on the environment, including forest ecosystems. In Sweden, in 2005, *T*. *piniperda* was found to begin flying approximately three weeks earlier than in the 1970s [96]. This observation is supported by meteorological data showing that the first possible flight date (maximum temperature > 12 °C) also occurred correspondingly earlier during the last decade than in the 1970 [96]. Additionally, very few specimens of *Tomicus minor* Hart. were captured in traps or found in fallen trees in the storm area [76]. Compared with the distribution reported in Lekander et al. [97], this may indicate the beginning of a retraction of the species in southern Sweden. Both of these observations may be the first signs of a response to a warmer climate, but it remains to be seen whether they were merely random occurrences or the beginning of a trend.

## 5. Conclusions

1. *C. piceae* has great economic significance as a secondary pest in the Holy Cross Mountains (Góry Świętokrzyskie). Its ability to colonise material in a lying position in conditions where the health of firs is impaired may lead to mass reproduction. The similar parameters obtained for the colonisation of natural traps by *C. piceae* mean that it is possible to use windfalls in order to limit its population numbers. Trap trees should be laid out as an exceptional measure, in the case of the reproduction of this bark beetle in very large numbers.

2. The physiological condition of trees and spatial specialisation are the main mechanisms that minimise competition between bark beetle species on fir. Differences in feeding preferences enable them to make effective use of the limited food resources that the stems provide.

3. An analysis of the dependence of the niche breadth on the stem features and on the parameters of bark beetle colonisation revealed the most significant correlation in the case of gallery density. The values of coefficients of determination were significant: for *C. piceae*, $R_{adj}^{2}\;$= 0.7669; for *P. curvidens*, $R_{adj}^{2}\;$= 0.8065; and for *P. spinidens*, $R_{adj}^{2}\;$= 0.8433. The mean relative estimation errors did not exceed 20%.

4. The results suggest the desirability of further research to obtain regression equations for firs growing in different regions of Europe. The presented regression equations should be calibrated and adapted to local conditions of the colonisation of firs by bark beetles. The development of local regression equations will increase the accuracy of the method. Analogous regression equations may also be derived for other cambiophagous and xylophagous species.

## Figures and Tables

**Table 1:** Parameters and basic statistics of Equations (4)–(7).

No. Equation	Parameters of Regression	Value of Parameter	*SE*	Value *t*-Statistics	*P*	$R_{adj}^{2}$
4	*a*_0_	0.3082	0.0286	10.7790	<0.001	0.6689
	*a*_1_	0.0005	<0.001	10.9641	<0.001	
5	*a*_0_	–0.5786	0.0830	–6.9684	<0.001	0.7669
	*a*_1_	0.1939	0.0139	13.9682	<0.001	
6	*a*_0_	0.1028	0.0161	6.3960	<0.001	0.8065
	*a*_1_	0.0106	0.0016	6.4416	<0.001	
	*b*_0_	0.4274	0.0495	8.6354	<0.001	
	*b*_1_	0.0006	0.0002	2.6094	0.0127	
7	*a*_0_	0.1126	0.0216	5.2157	<0.001	0.8432
	*a*_1_	0.0113	0.0039	2.9022	0.0068	
	*b*_0_	0.2629	0.0461	5.7043	<0.001	
	*b*_1_	0.0025	0.0004	6.7106	<0.001	

*SE*—standard error; *P*—probability level; $R_{adj}^{2}$—adjusted *R*-squared.

**Table 2:** Distribution of regression residuals.

No. Equation	Normal Distribution		Homogeneity of Variances	
	Shapiro–Wilk Test		White’s Test	
	*W*	*P*	*W*	*P*
5	0.9795	0.4095	4.5198	0.1044
6	0.9791	0.5964	1.8190	0.4027
7	0.9679	0.3890	2.9418	0.2297

*W*—value statistics; *P*—probability level.

## Data Availability

The data presented in this study are available on request from the corresponding author.
